# A definition for coaching in medical education

**Published:** 2019-11-28

**Authors:** Jeffrey Landreville, Warren Cheung, Jason Frank, Denyse Richardson

**Affiliations:** 1 Department of Emergency Medicine, University of Ottawa, Ontario, Canada; 2 The Royal College of Physicians and Surgeons of Canada, Ontario, Canada; 3 Department of Medicine, University of Toronto, Ontario, Canada

Consider world champion tennis player Roger Federer. As the number 1 ranked tennis player for a record 310 weeks, he represents one of the most successful professional athletes of our time. To what does he owe his success? While he undoubtedly possesses a remarkable amount of self-motivation, dedication, and athleticism; there is another factor to consider:he has a coach. In fact, he has a team of coaches who work on every aspect of his game with a common goal of performance enhancement. In a recent tribute to his coaches on social media, Federer wrote “Could never have been the oldest #1 without my team. Thank you to everyone who has helped me along the way”.^1^

Despite its wide application in other high-performance professions such as athletics, music, and business, coaching has only recently gained attention within medicine and medical education. The adoption of Competency-Based Medical Education (CBME) and emphasis on observation has led to increased use of coaching terminology within the medical education community. However, a clear definition of coaching is lacking, and people often use the term coaching interchangeably with related terms such as teaching and mentoring.^2^We need a clear operational definition of coaching in order to advance the use of coaching within medical education and to conduct meaningful research on this topic.

We believe, coaching is a process that guides a learner towards performance improvement. Coaching requires establishment of supervisor-learner rapport, setting of expectations, and observation of the activities that are being developed. Following observation, the supervisor and learner engage in a bi-directional conversation which leads to meaningful feedback and practical suggestions for performance improvement. Supervisors may document their conversations to provide a developmental trajectory over time. Educational researchers have previously defined coaching as a “one-to-one conversation focused on the enhancement of learning and development through increasing self-awareness and a sense of personal responsibility, where the coach facilitates the self-directed learning of the coachee through questioning, active listening, and appropriate challenge in a supportive and encouraging climate”.^3^However, thisdefinition is restrictive in its focus on self-directed learning. Further, it lacks important components of coaching such as learner observation for the purpose of providing meaningful feedback, and therefore limit the utility of this definition.

We consider coaching to be more than giving feedback. While feedback provides information about what the coach observed compared to an expected standard, coaching involves providing practical suggestions for improvement with the aim of enhancing learner performance at a specific activity. To go back to the sports analogy, Federer’s tennis coach would not simply tell him his forehand swing is incorrect. The coach would give specific suggestions for improvement such as adjusting the position of the body during the forehand swing.

Coaching in a medical education context has been conceptualized into two types.^5^ Coaching in the Moment (CiM) refers to coaching that occurs between a clinical teacher and learner within the clinical practice environment. CiM includes observation, feedback and actionable suggestions for performance improvement. CiM in our Federer scenario is where one of Federer’s tennis coaches watches his swing during a practice session.

The second type of coaching is Coaching over Time (CoT). CoT refers to coaching that occurs between a supervisor and learner outside of the clinical environment. Observation is based primarily on the learner’s performance data that has been collected and compiled. Similar to CiM, feedback and suggestions for performance improvement remain key components. CoT is imperative to guiding learners in their clinical progress and promoting their development ascompetent clinicians. CoT in our Federer scenario is when Federer meets with his coachevery few months to review his on-court performance metrics and determine how he can improve them.

Twoterms that people frequently use interchangeably with coaching are teaching and mentoring. Teaching typically follows a directive approach where the goal of the interaction is acquisition of knowledge, skills, or attitudes on the part of the recipient. Teaching differs from coaching as teaching does not rely on observation or conversation. Mentoring refers to a confidential, non-judgmental relationship between two individuals with the ultimate goal of encouraging the mentee to take charge of their own development.^4^ Mentoring differs from coaching as mentoring is not focused on performance improvement but rather seeks to provide guidance and support.

Coaching is gaining popularity within medicine and medical education yet there is a conceptual tension surrounding how it differs from teaching and mentoring. At present, coaching within medical education is lacking a clear operational definition that is relevant to both education researchers and front-line clinicians. We have advanced one such definition emphasizingcoaching as a process that guides a learner towards performance improvement.We look forward to productive debate.
